# Traumatic stapediovestibular luxation: two contrasting cases highlighting the importance of early treatment decision: A case report and literature review

**DOI:** 10.1097/MD.0000000000049594

**Published:** 2026-07-03

**Authors:** Saki Takahama, Kohei Yamahara, Takayuki Okano, Ichiro Tateya

**Affiliations:** a Department of Otolaryngology-Head and Neck Surgery, School of Medicine, Fujita Health University, Toyoake, Aichi, Japan; b Department of Otolaryngology-Head and Neck Surgery, School of Medicine, Fujita Health University Bantane Hospital, Toyoake, Aichi, Japan.

**Keywords:** corticosteroids, hearing loss, stapes footplate, traumatic stapediovestibular luxation, vertigo

## Abstract

**Rationale:**

Traumatic stapediovestibular luxation is a rare but potentially devastating condition that may cause severe vertigo and hearing loss. Optimal management remains controversial because surgical manipulation around the stapes carries the risk of additional inner ear damage, whereas some patients recover with conservative treatment alone.

**Patient concerns:**

Two patients presented with vertigo and hearing impairment after accidental penetrating ear trauma caused by cotton swabs.

**Diagnoses:**

High-resolution computed tomography demonstrated stapes footplate invagination into the vestibule in both cases.

**Interventions:**

In Case 1, early surgical repositioning of the stapes was performed on the third day after injury because of complete footplate invagination accompanied by bone-conduction hearing loss and severe vertigo. In Case 2, conservative management was selected because bone-conduction thresholds were preserved and vestibular symptoms improved spontaneously.

**Outcomes:**

In Case 1, postoperative recovery included rapid resolution of vertigo and improvement of bone-conduction thresholds, particularly at low frequencies. In Case 2, vestibular symptoms resolved without surgery, although a mild residual air-bone gap remained.

**Lessons:**

Bone-conduction deterioration, degree of footplate displacement, severity of vertigo, and depth of invagination on high-resolution computed tomography appear to be key factors for treatment selection. Conservative management may be safely continued in selected cases with mild posterior partial luxation and stable bone-conduction, whereas early surgical decision-making is essential when surgery is indicated to optimize vestibular and auditory outcomes.

## 1. Introduction

The stapes footplate can become dislocated and invaginated into the vestibule after penetrating trauma from objects such as earpicks or cotton swabs or during otologic surgical procedures. Although traumatic stapediovestibular luxation is a relatively rare condition, it can lead to intense vertigo and severe hearing loss. Therefore, determining an appropriate management strategy is crucial. Typically, management begins in the early post-traumatic period with conservative measures such as systemic corticosteroids and strict bed rest, after which the indications for surgical intervention are considered.^[1]^ However, surgical manipulation around the stapes and oval window carries a substantial risk of inducing additional inner ear damage, which may lead to the emergence or exacerbation of postoperative sensorineural hearing loss.^[1–3]^ Conversely, several patients have achieved resolution of vestibular symptoms and hearing improvement with conservative management alone.^[4–6]^ Consequently, no specific protocol or universally accepted criteria currently exist regarding the optimal timing of surgery or whether exploratory intervention is mandatory in every case.^[2]^

Herein, we describe 2 cases of traumatic stapediovestibular luxation: 1 managed with early surgical intervention and the other that followed a favorable course with continuous conservative care. A distinctive feature of both cases was early decision-making regarding the management strategy based on a comprehensive assessment of high-resolution computed tomography (HRCT) results, pure-tone audiometry (PTA), nystagmus findings, and clinical symptoms. The timing of the management decision substantially influenced the clinical outcomes. Therefore, we discuss the conditions under which conservative management can be safely selected. Furthermore, we discuss the appropriate time window for surgical intervention via a comparison with previously reported cases of radiologically confirmed stapedial luxation.

## 2. Case presentation

### 2.1. Case 1

**Figure 1.**
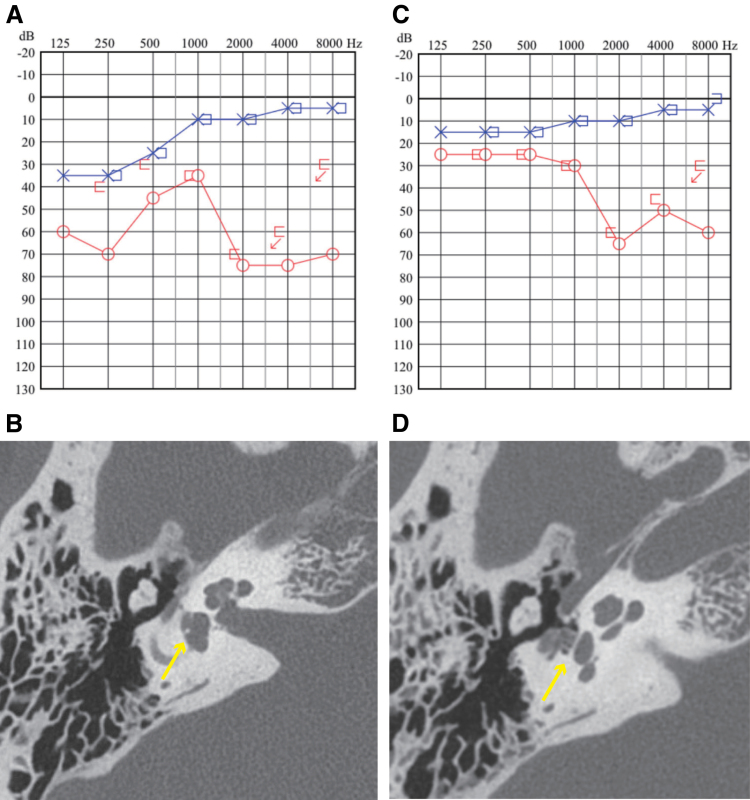
Preoperative and postoperative CT images and audiograms of Case 1. (A) Preoperative PTA. (B) Preoperative axial CT images of the temporal bone. The stapes footplate was completely invaginated into the vestibule (yellow arrow). (C) Postoperative PTA. (D) Postoperative axial CT images of the temporal bone. The stapes footplate was successfully repositioned in its anatomical place (yellow arrow). The arrow points to the cartilage placed on the oval window. The incus columella (arrowhead) was placed on the cartilage (arrow). The star indicates elevated TM for all figures. CT = computed tomography, PTA = pure-tone audiometry, TM = tympanic membrane.

**Figure 2.**
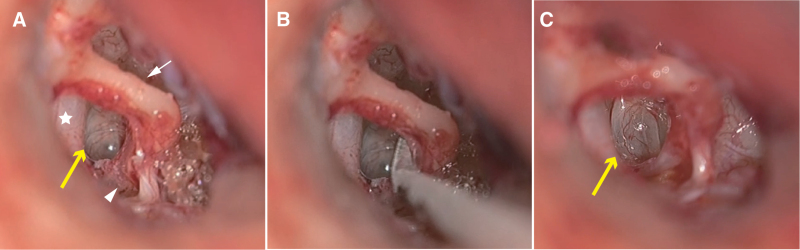
Intraoperative findings. (A) The stapes footplate was invaginated as seen on imaging, without associated ossicular disarticulation. The yellow arrow points to the invaginated stapes footplate. The white arrow points to the incus long crus. The arrowhead points to the stapes posterior crus. The star points to the facial canal. (B-C) The stapes was meticulously and carefully repositioned to the oval window using a hook.

A 49-year-old woman had an accidental penetrating injury to the right ear while cleaning it with a cotton swab at night. This injury was triggered by the patient’s dog jumping on her. The patient experienced immediate hearing loss and dizziness, and visited the emergency room on the same day. Physical examination showed a perforation in the posterosuperior quadrant of the right tympanic membrane (TM) and horizontal spontaneous leftward nystagmus, suggestive of right-sided paralytic nystagmus. Inner ear damage linked to TM perforation was suspected, and the patient was urgently admitted and started on bed rest, intravenous corticosteroids, and anti-vertigo medications. On the day of admission, PTA and HRCT were conducted. PTA detected right mixed hearing loss (Fig. 1A). Although HRCT revealed no ossicular chain interruption, the stapes footplate had completely invaginated into the vestibule (Fig. 1B and Fig. S1A, Supplemental Digital Content 1). By the second day following injury, conservative management began to show effects. Gaze nystagmus disappeared, and although some residual dizziness persisted, it showed a recovery trend. Although conservative management produced partial improvement by the second day after injury, bone-conduction hearing loss persisted on PTA, and HRCT demonstrated complete invagination of the stapes footplate into the vestibule. These findings raised concern that spontaneous sealing of the oval window might be difficult with conservative treatment alone. Therefore, early surgical repositioning was performed on the third day after trauma. During surgery, we confirmed that the stapes was invaginated, as noted on imaging, without associated ossicular disarticulation (Fig. 2A). The stapes was meticulously repositioned into the oval window using a hook (Figs. 2B and 2C). The footplate was sealed with fat and fixed using fibrin glue. Postoperatively, the dizziness completely subsided 1 day following surgery. Bed rest was lifted 7 days after surgery without worsening the vertigo, and the patient was discharged 10 days following surgery. At the 3-month follow-up, PTA revealed recovery of the bone-conduction thresholds, especially at lower frequencies, and the air-bone gap disappeared across all frequencies (Fig. 1C). HRCT showed that the stapes was successfully repositioned in its anatomical location, while soft tissue shadows remained around it (Fig. 1D and Fig. S1B, Supplemental Digital Content 1).

### 2.2. Case 2

**Figure 3.**
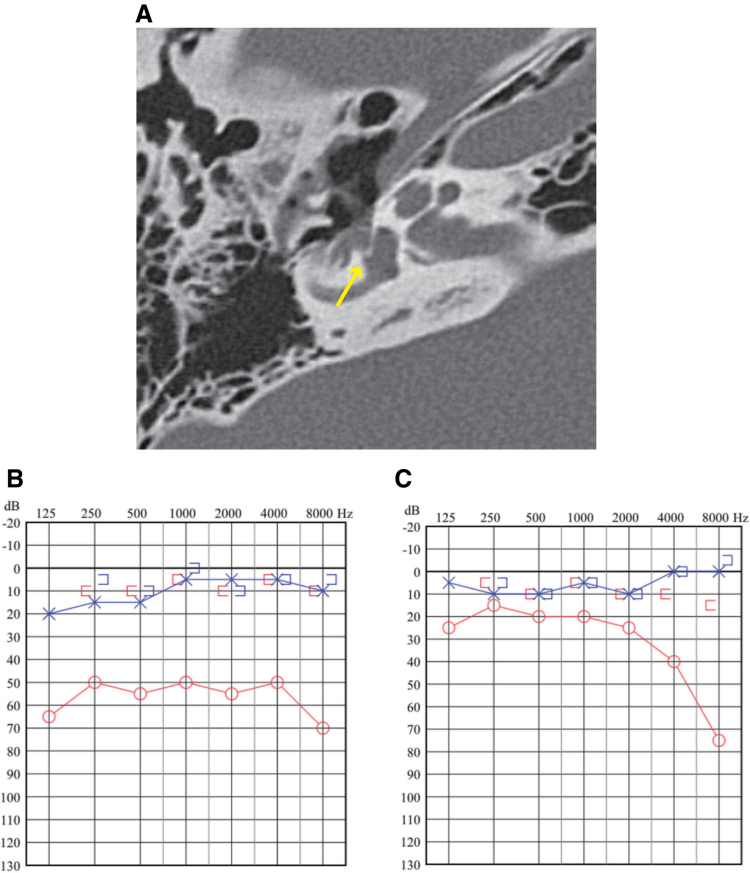
CT images and audiograms of Case 2. (A) Audiogram before treatment. (B) CT image. A partial invagination involving only the posterior aspect of the stapes footplate was seen (yellow arrow). (C) Audiogram after conservative treatment. CT = computed tomography.

A 25-year-old man accidentally thrust a cotton swab deep into his right ear canal while cleaning it. The patient immediately experienced dizziness, nausea, hearing loss, and tinnitus and presented to the emergency room on the same day. Physical examination revealed a TM perforation and leftward horizontal nystagmus. PTA revealed conductive hearing loss with no elevation in bone-conduction thresholds (Fig. 3B). HRCT showed incudostapedial joint disarticulation and partial invagination involving only the posterior aspect of the stapes footplate (Fig. 3A and Fig. S1C, Supplemental Digital Content 1). The patient was admitted for bed rest and intravenous treatment with corticosteroids and anti-vertigo agents. We decided to continue conservative management without surgical intervention, given that his vestibular symptoms exhibited an improving trend by day 5 and bone-conduction thresholds remained stable. Restrictions were lifted on day 6. Although mild dizziness persisted, the patient was able to walk and tolerate oral intake and was discharged on day 13. The TM perforation spontaneously closed within 1 month. At the 2-month follow-up, PTA revealed a right hearing level of 15.0 dB. Although a mild air-bone gap persisted at higher frequencies (Fig. 3C), the patient’s vestibular symptoms completely resolved.

## 3. Discussion

Penetrating trauma through the external ear canal or, sometimes, indirect trauma causes stapediovestibular luxation. The majority of reported cases in the literature are from Japan, likely because Japanese people are frequent ear pick users.^[7]^ Typically, penetrating ear trauma caused by earpicks or cotton swabs can injure the middle ear structures through perforation of the TM. This potentially results in ossicular chain disruption, internal stapes dislocation (invagination into the vestibule), or external dislocation to the tympanic cavity, with clinical presentations markedly varying between cases.^[8,9]^

Although several authors recommend early surgical correction for this condition, no specified or standardized treatment protocol exists for surgical intervention, and controversy remains regarding whether exploratory surgery is indispensable in every case.^[2,9]^ Surgical management requires procedures around the stapes and oval window. However, stapes manipulation carries the risk of causing additional inner ear damage, requiring meticulous and extremely careful operative techniques.^[1,2,10]^

In contrast, inner ear windows possess the capacity for spontaneous repair.^[4,11]^ In fact, several cases have been reported in which patients managed conservatively achieved resolution of vestibular symptoms and recovery of sensorineural hearing function.^[5,6]^ Consequently, clinicians often face a dilemma when deciding whether to proceed with surgical intervention in cases revealing a tendency toward recovery under conservative management. Furthermore, no single definitive treatment choice exists as the safety of ossicular surgery depends on the surgeon’s level of expertise, experience, and institutional conditions.^[1]^ Rather, what is clinically critical is to consider these factors and clarify the criteria to determine cases for which conservative observation can be safely selected.

Table 1 summarizes the clinical courses of previously reported cases of radiologically confirmed stapediovestibular luxation.^[1–6,8,9,11–24]^ In most cases that progressed from conservative treatment to surgery, the stapes was completely depressed or deeply invaginated into the vestibule. Moreover, bone-conduction thresholds deteriorated or vestibular symptoms worsened during the course of conservative therapy. Conversely, a common feature among cases successfully managed with conservative treatment is that the degree of stapes luxation is relatively mild, bone-conduction thresholds remain stable without further deterioration, and vestibular symptoms show early improvement. Although dizziness or an elevated bone-conduction threshold may be present at the time of injury, this eventually results in spontaneous sealing of the fistula.^[4–6]^ These results suggest that the degree of dislocation on imaging may serve as a predictive factor of resistance to conservative management. In addition, the timing of management decisions is crucial. In some patients who eventually underwent surgery after the initial observation, surgical exploration was prompted by the fact that the symptoms or hearing thresholds failed to improve or even deteriorated during conservative management (Table 1).^[2,6,8,9,11,13,15–19,22,23]^ This indicates that while initiating conservative management is common, a prompt change in strategy is necessary if the progression of symptoms and hearing levels indicates the requirement for surgery. In other words, in the management of this disease, assessing whether there is a tendency toward improvement within a short period and identifying cases that require surgery without delay can substantially affect the clinical outcome.

**Table 1:** Cases of radiologically confirmed stapediovestibular luxation.

Author	Cause	CT Findings or intraoperative stapes status	PTA (pretreatment)	Preoperative Symptom Improvement	Interval to Surgery	Management Strategy	Clinical Outcome
Chujo et al^[12]^	Indirect head trauma	Fracture at posterior crus and footplate; dislocated posteriorly.	CHL	Partial. Vertigo subsided, but HL persisted.	2 months	Surgical: SD + malleovestibulopexy	Vertigo resolved; hearing improved
Ren et al^[13]^	Earpick	Footplate protrusion into the vestibule and IS joint disarticulation.	Severe mixed HL (83.3 dB).	None	4 months	Surgical: RS	Vertigo resolved; hearing improved to 66.7 dB.
Ginat et al^[14]^	Stick	Extensive pneumolabyrinth and complete luxation into the vestibule and intact ossicular chain	Mixed HL (80 dB).	N/A	N/A	Surgical: SD	Complete resolution of vertigo and disequilibrium.
Nakajima et al.^[8]^ Case 1	Earpick	Pneumolabyrinth and stapes invagination into the vestibule.	Moderate mixed HL.	None. Dizziness worsened and BC thresholds deteriorated by day 4.	5 days	Surgical: RS and OS	Vertigo resolved; hearing improved
Hatano et al^[2]^	Earpick	Pneumolabyrinth and deep stapes depression into the vestibule.	Severe mixed HL (110 dB).	None	11 days	Surgical: Stapes left in vestibule, OS, and columella insertion between OW and TM	Vertigo resolved; hearing improved to moderate mixed HL (51 dB).
Yamahara et al^[1]^	Barotrauma (nasal blowing)	Pneumolabyrinth in vestibule/semicircular canal/cochlea; stapes depression into vestible.	Severe mixed HL (PTA 100 dB).	Partial. Vertigo improved, but hearing persisted.	7 days	Surgical: SD, OS with cartilage, and incus columella between OW and TM	Vertigo resolved; ABG closed to within 10 dB.
Paramasivam et al^[15]^	Stick	Presence of air pocket within the membranous labyrinth, complete luxation of the stapes, and stapes suprastructure fracture	Severe HL (> 90 dB).	None	2 months	Surgical: SD + RS	Relief of dizziness; no improvement in hearing.
Ishida et al^[16]^	Tip of comb	Footplate protrusion into the vestibule, IS joint disarticulation, and fraction of stapes crura	Severe mixed HL (87.5 dB).	None	8 days	Surgical: SD + connective tissue between OW and incus	Vertigo resolved in 3 days; hearing improved to 23.8 dB.
Yamasoba et al^[3]^	Earpick	Deep stapes depression into vestibule and pneumolabyrinth.	Mild HL (PTA 30 dB).	N/A Prompt surgical intervention performed.	Immediate (0 days)	Surgical: SD + cartilage columella between OW and TM	Dizziness resolved in 7 days; hearing stabilized at moderate combined HL (45 dB).
Ederies et al^[17]^	Eyeglass temple	Anterior rotation (> 90°) and depression of the stapes and fractured footplate/suprastructure	Severe mixed HL (BC 39 dB/ AC 88 dB).	None	2 months	Surgical: Partial retrieval of stapes and OS	Resolution of vertigo; hearing remained stable.
Wang et al^[18]^ Case 11	Branch	Stapes depression into vestibule.	Conductive HL (PTA 57.5 dB).	None. Vertigo recurred after initial conservative management.	3 months	Surgical: SD and piston reconstruction.	Resolution of vertigo; significant hearing improvement (PTA 32.5 dB).
Lao and Niparko^[5]^	Tree branch	pneumolabyrinth within the vestibule and luxation of the stapes	Moderate to profound mixed HL.	Yes. Hearing and pneumolabyrinth improved with bed rest and steroids.	−	Conservative:	Hearing recovered to near-normal; pneumolabyrinth resolved.
Comacchio et al^[19]^	Earpick	partial luxation of the stapes, stapes footplate fracture, and extensive pneumolabyrinth	Severe mixed HL (BC 64 dB/ AC 91 dB).	None	N/A (Revision at > 2 yrs)	Surgical: Stapes left in place and OS	Positional vertigo resolved; no improvement in hearing.
Kagoya et al^[20]^	Traffic accident (Indirect)	Partial luxation of the staps, stapes superstructure dislocated outward/downward.	Conductive HL (PTA 60 dB).	Partial. Dizziness gradually subsided over 2 months.	16 months	Surgical: OS and cartilage columella between OW and incus	Hearing recovered significantly (PTA 23 dB).
Khoo et al^[4]^	Earpick	Complete invagination of the stapes into vestibule; deeply depressed and inverted	Mixed HL.	Yes. Sensorineural function recovered after 1 month.	−	Conservative: Due to high surgical risk and spontaneous sealing.	Sensorineural hearing recovered; conductive HL persisted.
Lee and Lee^[11]^	Penetrating trauma	Complete invagination of the stapes into vestibule (deeply depressed).	Conductive HL (AC 49 dB/ BC 16 dB).	None	Immediate	Surgical: SD and piston wire reconstruction.	Vertigo rapidly subsided; hearing improved to normal range.
Oulghoul et al^[21]^	Q-tip	Complete stapes intrusion into vestibule and pneumolabyrinth.	Moderate conductive HL.	N/A.	2 days	Surgical: SD and Teflon piston reconstruction.	Resolution of vertigo; hearing improved (mild CHL).
Nishiike et al^[22]^	Tip of comb	Partial invagination of the stapes into vestibule (slightly depressed).	Mixed HL (58.3 dB).	None.	4 days	Surgical: RS and ossiculoplasty.	Vestibular symptoms resolved; mixed HL persisted (76.7 dB).
Hidaka et al^[6]^ Case 1	Chopstick	Complete invagination of the stapes into vestible; inverted and luxated..	Near-total deafness.	None. Symptoms failed to improve over 1 week.	32 days	Surgical: SD and gentamicin injection.	Vertigo subsided; hearing remained deaf.
Hidaka et al^[6]^ Case 2	Earpick	Air bubble within the vestibule.	Mixed HL (PTA 36.7 dB).	Yes. Vertigo subsided in 5 days; hearing improved in 1 week.	−	Conservative:	Hearing improved to 18.3 dB at 2 months.
Hidaka et al^[6]^ Case 3	Earpick	Air bubble within the vestibule.	Mixed HL (PTA 76.7 dB).	Yes. Vertigo subsided in 7 days; hearing improved in 8 days.	−	Conservative:	Hearing improved to 35 dB at 2 months.
Snelling et al^[23]^	Hair band	Complete invagination; footplate pushed through OW.	Conductive HL (initial); Mixed HL (at 3 month).	None. Hearing deteriorated and rotatory vertigo emerged.	3 months	Surgical: Perilymph leak repair with fat plug.	“Dead ear” (hearing loss deteriorated); tinnitus resolved.
Bogaerts et al^[9]^	Head massager	Partial invagination of the stapes into vestibule (specifically the posterior crus).	Mixed HL (PTA 38 dB).	None.	9 days	Surgical: RS with silastic “stapedial strut.”	Vertigo/tinnitus resolved; BC thresholds stabilized.

ABG = air-bone gap, BC = bone conduction, CHL = conductive hearing loss, HL = hearing loss, N/A = not available, OS = oval window sealing, OW = oval window, PTA = Pure-tone audiometry, RS = repositioning of the stapes, SD = stapedectomy, TM = tympanic membrane.

Although both cases in this report involved traumatic stapes dislocation, they showed markedly varying clinical pathologies, leading to a clear contrast in management strategies and clinical outcomes. In Case 1, the entire footplate was deeply invaginated into the vestibule, accompanied by an elevation in bone-conduction thresholds and severe vestibular symptoms. Although initial conservative management resulted in a certain degree of improvement, such as a reduction in nystagmus, the presence of a relatively large bone-conduction hearing loss and a high degree of stapedial invagination suggested that spontaneous sealing of the oval window was unlikely with conservative management alone. Consequently, surgical intervention was performed on the third day after injury. This decision is consistent with those of previous reports, in which cases of complete dislocation often showed progressive hearing deterioration or worsening symptoms during conservative observation.^[8]^ Notably, in this case, vestibular symptoms rapidly resolved postoperatively and bone-conduction thresholds were restored, especially at lower frequencies. Although improvement in bone-conduction hearing levels is rare,^[2,8]^ our findings suggest that bone-conduction recovery is possible with timely surgical intervention. Thus, in cases where surgery is indicated, early determination of the management strategy is important for the control of vestibular symptoms and the prognosis of sensorineural hearing function. Conversely, Case 2 involved only a slight medial displacement of the posterior aspect of the stapes footplate. Furthermore, the bone-conduction thresholds remained within the normal range, and vestibular symptoms spontaneously resolved over a short period. CT results lacked evidence of a deep invagination or definitive signs of an extensive perilymphatic fistula, and a favorable outcome was achieved via conservative management. The absence of bone-conduction threshold elevation in this case suggests that the membranous labyrinth remained intact with minimal perilymph leakage, allowing early spontaneous sealing of the inner ear window.

Notably, a partial dislocation localized in the posterior part of the stapes footplate may be less likely to cause substantial bone-conduction hearing loss. Bogaerts et al reported a case wherein the posterior part of the stapes was depressed, similar to ours, where the preoperative bone-conduction thresholds were only mildly elevated at 1 to 2 kHz.^[9]^ Additionally, the case described by Chujo et al involving isolated posterior displacement did not show any elevation in bone-conduction thresholds.^[12]^ These findings suggest that inner ear damage may remain relatively minor in cases of partial luxation limited to the posterior footplate. The relationship between the footplate invagination site and inner ear symptoms warrants further discussion from an anatomical perspective. Anatomical studies of the human temporal bone have reported that the saccule is located near the anterior and central portions while the utricle is most closely situated to the posterior aspect of the footplate.^[25–27]^ Additionally, morphometric research associated with stapedotomy has shown that the distance from the anterior margin of the footplate to the saccule is < 1 mm, and the distance from the inferior margin to the cochlear duct is as small as 0.2 mm in the shortest case.^[26]^ These results suggest that a stapedial invagination directed toward the anterior or anteroinferior region is more likely to affect delicate membranous structures, including the saccule and hook regions of the cochlear duct. Based on the anatomical background discussed, the primary factor contributing to the mild clinical course and spontaneous recovery in Case 2 was likely that the invagination was localized to the posterior aspect and was shallow. This avoided substantial impact on the delicate anterior and anteroinferior structures. Contrastingly, in Case 1, the entire footplate was deeply invaginated into the vestibule, suggesting that the widespread involvement of inner ear structures, including the anterior elements, contributed to the severe vestibular symptoms and elevation of bone-conduction thresholds. However, posterior footplate dislocation does not always guarantee a favorable prognosis. Bogaerts et al reported that vestibular symptoms persisted despite shallow posterior invagination, eventually requiring surgical intervention.^[9]^ Conversely, Khoo et al documented a case in which, despite complete luxation of the stapes deep into the vestibule, the patient achieved resolution of vertigo and recovery of sensorineural function via conservative management.^[4]^ These reports highlight the danger of determining prognosis or management strategy based solely on radiological results. Furthermore, a “successful” outcome via conservative management does not necessarily imply that surgery is not indicated. In Case 2, a mild air-bone gap persisted following treatment. Thus, the fact that further closure of the air-bone gap might have been achieved if surgical reconstruction was performed cannot be ruled out.

Based on our 2 cases and a review of the literature (Table 1), we considered 4 critical factors when determining the management strategy: depth and anatomical direction of stapedial invagination on HRCT, degree of footplate displacement (partial vs complete luxation), presence of bone-conduction hearing loss, and severity and early course of vestibular symptoms. Patients with mild displacement, preserved bone-conduction, and rapid improvement of vestibular symptoms are likely to follow a safe course under conservative management. This is particularly true for partial posterior dislocations with shallow invaginations, as shown in Case 2, providing a useful guideline for cases in which surgical risks must be carefully weighed or for surgeons with limited experience. Furthermore, management decisions must be made promptly depending on the situation because cochleovestibular dysfunction can worsen during conservative management. Thus, defining the management strategies early is crucial for conservative observation or surgical exploration. The early response to strict bed rest and systemic corticosteroid therapy is also clinically important, particularly within the first 24 to 48 hours, because improvement of vertigo or nystagmus may support continued conservative observation in selected patients. However, this response should be interpreted as an adjunctive dynamic factor rather than an independent determinant. Even when vestibular symptoms partially improve, persistent bone-conduction deterioration or complete or deep footplate invagination on HRCT may still indicate substantial inner ear injury and should prompt consideration of early surgical intervention.

This study has several limitations. First, it included only 2 cases, and the proposed factors for treatment selection should therefore not be regarded as definitive criteria. Second, quantitative vestibular assessments, including video head impulse test, were not performed. Third, serial audiometric data during the acute phase were limited. Fourth, HRCT could demonstrate the position of the stapes footplate but could not directly assess membranous labyrinth injury or the degree of perilymph leakage. Finally, the indication for early surgery may vary depending on the surgeon’s experience and institutional conditions.

We anticipate that the treatment algorithm for traumatic stapediovestibular luxation will be further refined based on a deeper understanding of its pathophysiology with the continued accumulation of reported cases.

## 4. Conclusions

Traumatic stapediovestibular luxation presents with highly variable clinical features, and its optimal management remains controversial. Our 2 cases show that early determination of the treatment strategy based on bone-conduction thresholds, degree and depth of footplate displacement on HRCT, and severity of vestibular symptoms is important. Conservative management may safely be continued in selected cases with mild posterior partial luxation and preserved bone-conduction; however, timely surgical intervention should be considered in cases of bone-conduction deterioration or deep invagination. Early surgical decision-making may control vestibular symptoms and contribute to the recovery of sensorineural hearing.

## Author contributions

**Conceptualization:** Saki Takahama, Kohei Yamahara, Takayuki Okano.

**Data curation:** Kohei Yamahara, Takayuki Okano.

**Formal analysis:** Kohei Yamahara.

**Supervision:** Kohei Yamahara, Takayuki Okano, Ichiro Tateya.

**Writing** – **original draft:** Saki Takahama.

**Writing** – **review & editing:** Kohei Yamahara.


