# Supplementary figures and images for: Traumatic stapediovestibular luxation: two contrasting cases highlighting the importance of early treatment decision: A case report and literature review
Source: Medicine (Baltimore). 2026 Jul 3;105(27):e49594. doi: 10.1097/MD.0000000000049594 (PMC13337014; doi:10.1097/MD.0000000000049594)

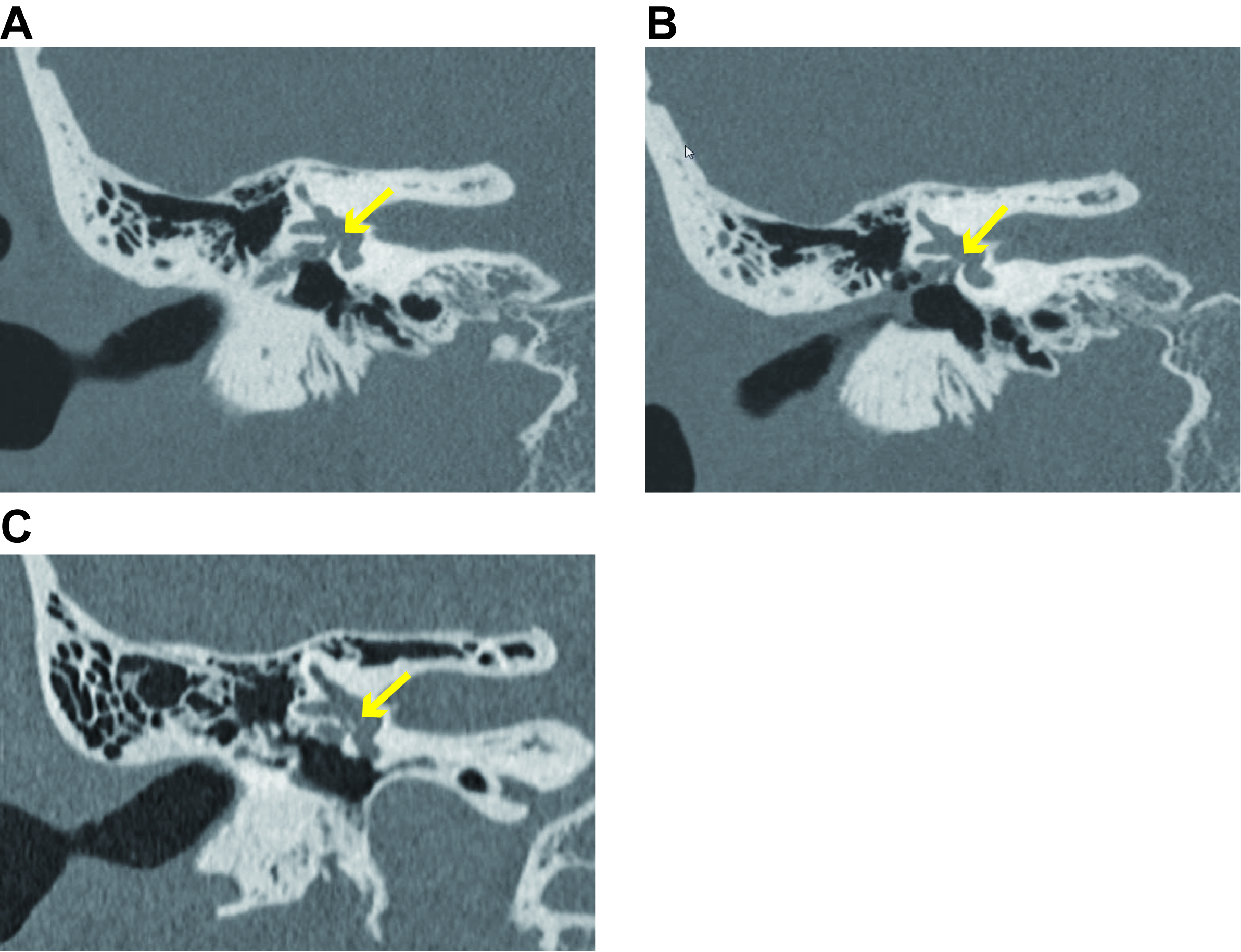

Supplement: Supplementary file 1 [file medi-105-e49594-s001.tif]
